# A Longitudinal Study of Authoritative Parenting, Juvenile Delinquency and Crime Victimization among Chinese Adolescents

**DOI:** 10.3390/ijerph17041405

**Published:** 2020-02-21

**Authors:** Ruoshan Xiong, Spencer De Li, Yiwei Xia

**Affiliations:** 1 Department of Sociology, University of Macau, Macau 999078, China; xiongruoshan1991@163.com (R.X.); spencerli@um.edu.mo (S.D.L.); 2 School of Law, Southwestern University of Finance and Economics, Chengdu 611130, China

**Keywords:** Authoritative parenting, delinquent peer association, mental health, delinquency, crime victimization

## Abstract

Empirical research on the relationship between authoritative parenting and crime victimization has been sparse, although this style of parenting has been identified as an effective parenting practice for inhibiting offending behavior among children and adolescents. The current research aims at filling this gap by examining the influences of authoritative parenting on juvenile delinquency and crime victimization, as well as the mechanisms connecting the processes. Using two-wave survey data collected from a probability sample of 1066 Chinese adolescents, the current study employed a structural equation modeling analysis to test the relationships. The results indicated that authoritative parenting negatively predicted juvenile delinquency and crime victimization. Further, adolescent mental health problems and delinquent peer association partially mediated the influence of authoritative parenting on delinquency, while adolescent mental health problems, delinquent peer association, and juvenile delinquency fully mediated the relationship between authoritative parenting and crime victimization. The results also showed that juvenile delinquency positively predicted future crime victimization. Overall, this study demonstrated that authoritative parenting operated as a protective factor against juvenile delinquency and crime victimization.

## 1. Introduction

The issue of adolescent crime victimization has drawn considerable attention in empirical research [1]. Studies have shown that more than 70% of adolescents had been exposed to at least one form of victimization in the preceding year [2,3]. Research has linked the high rate of prevalence to parenting styles, as evidence has shown that effective parenting style could protect adolescents from risky relationships and situations [4,5]. As an important correlate of delinquency in criminological research, authoritative parenting style has been found to operate as an effective parenting style in inhibiting juvenile delinquency [6,7,8,9]. However, unlike its impact on delinquent behavior, the influence of authoritative parenting on crime victimization has only been sparsely investigated in empirical research conducted in western and eastern societies. The current study aims to narrow this research gap. The main objectives of the study are to examine the influence of authoritative parenting on adolescent crime victimization and the mechanisms through which such influence takes place. We build a theoretical model linking authoritative parenting to crime victimization, which incorporates both a direct effect and several indirect effects through mental health problems, delinquent peer association, and delinquency. These mediators are selected because they have been shown to be potentially connected to parenting styles as response variables and as predictors of crime victimization in prior research [8,10,11].

The research questions are particularly pertinent to victimization studies in China where adolescent victimization has become an increasingly severe public health issue [2]. Chinese parents have a tendency of prioritizing children’s academic achievement over their psychological and social wellbeing [12]. This preference may be due, at least in part, to the long-held cultural belief that socially-defined achievement (e.g., doing well in school) transcends individual needs [13]. In the Chinese context, parents often employ strict disciplinary strategies to train children to adhere to socially desirable behavior and place a special emphasis on their academic and social achievement [12,13,14]. Correspondingly, the existing research on parenting styles in Chinese society has focused predominantly on their educational attainment. The lack of attention to children’s psychosocial wellbeing underscores the importance of identifying the critical role of parenting style in adolescent non-academic developmental outcomes as well as the social and psychological processes that shape the role.

### 1.1. Authoritative Parenting, Juvenile Delinquency, and Crime Victimization

A substantial body of sociological and psychological studies examining the relationship between parenting styles and children’s psychosocial development have corroborated that the most effective parenting combines demandingness with responsiveness [8,9,15,16]. Demandingness is defined as parents’ capability to provide close monitoring and willingness to discipline and confront the child who is in breach of rules. Responsiveness is referred to as the extent to which parents intentionally foster the child’s individuality and assertiveness through being supportive and responsive to child’s demands. Baumrind’s parenting style perspectives referred to such types of parenting as authoritative parenting [6,15,17]. The success of authoritative parenting lies in the fact that it can strike a balance between control (demandingness) and support (responsiveness), which fosters children’s internalization of various norms and values that are essential to their psychosocial development [17]. Authoritative parenting has been most frequently identified in prior research as an effective parenting style to promote child development and inhibit problem behavior [15,17,18], although emerging evidence indicates that indulgent parenting might also be conducive to positive developmental outcomes in specific cultural settings [19,20].

Consistent with the propositions of the parenting style perspectives, extensive literature has suggested that authoritative parenting is significantly and negatively related to juvenile delinquency. For example, in a study of 1355 juvenile offenders, Steinberg and his colleagues found that those who described their parents as authoritative were less likely to have externalizing problems compared to their counterparts exposed to other styles of parenting [21]. This finding is in line with Okorodudu’s study, which showed that authoritative parents were more likely to protect their children from engaging in delinquent behavior [22].

The relationship between authoritative parenting and adolescent crime victimization has received limited attention. However, prior research has demonstrated that specific dimensions of authoritative parenting such as close supervision, high levels of parental involvement, and support serve as protective factors against peer victimization [23]. Analyzing two waves of data collected from a sample of serious juvenile offenders, Tillyer and colleagues recently found that the combination of support and control, which was comparable to authoritative parenting, decreased adolescents’ risk for crime victimization [16]. Based on theories and empirical evidence reviewed in this section, we therefore hypothesize the following:

**Hypothesis** **1** **(H1):** *Authoritative parenting is negatively related to juvenile delinquency.*

**Hypothesis** **2** **(H2):** *Authoritative parenting is negatively related to adolescent crime victimization.*

### 1.2. The Mediating Role of Delinquent Peer Association

Research has suggested that an important way in which authoritative parenting facilitates children’s well-being is by promoting their affiliation with prosocial peer groups and steering them away from deviant subculture groups [24,25]. Authoritative parents often adopt a range of strategies in their childrearing to influence their children’s choices of peer associates, including selecting the neighborhood where the family lives [26], encouraging children’s participation in conventional types of activities [27], carefully selecting the schools they attend [28], and inviting their friends to participate in family activities [29].

Prior studies have suggested that authoritative parenting is significantly related to adolescents’ peer association. For example, Henry, Tolan, and Gorman-Smith found that adolescents growing up in families characterized by consistent discipline and warm interpersonal relationships had fewer delinquent peers compared to their counterparts who were subject to low parental support and inconsistent discipline [30]. Empirical evidence has shown that adolescents who are strongly attached to their parents are more likely to follow parents’ advice regarding friend choices and are more willing to share with their parents their peer groups, rendering them less susceptible to delinquent peer groups [31].

Sociological theories contend that delinquent peer association is a strong predictor of juvenile delinquency [32]. In an analysis of data collected from a representative sample of 2496 Chinese adolescents, Liu et al. found that delinquent peer association acted as a critical link between parenting practices and juvenile delinquency [33]. Empirical evidence has suggested that adolescents who associate with delinquent peer groups are more likely to be isolated and rejected by mainstream groups, which further encourages their affiliation with delinquent peer groups [34]. Whitbeck and his colleagues found that adolescents exposed to delinquent subculture groups were placed in high-risk situations, which heightened their vulnerability to victimization [35]. Similarly, a recent longitudinal study of 2168 South Korean adolescents by Hong, Kim, and Piquero found that delinquent peer association placed adolescents in jeopardy of peer victimization [36].

Based on evidence provided in prior research, we expect that delinquent peer association operates as a critical mediator linking authoritative parenting to adolescent delinquency and crime victimization. We propose the following hypotheses:

**Hypothesis** **3** **(H3):** *Authoritative parenting decreases adolescents’ delinquent peer association, leading to a lower likelihood of juvenile delinquency.*

**Hypothesis** **4** **(H4):** *Authoritative parenting decreases adolescents’ delinquent peer association, resulting in a lower likelihood of crime victimization.*

### 1.3. The Mediating Role of Mental Health Problems

In addition to delinquent peer association, mental health problems also serve as one of the mechanisms linking authoritative parenting, juvenile delinquency, and crime victimization.

Parenting style perspectives propose that authoritative parenting is significantly related to adolescent psychological wellbeing [37]. Authoritative parenting’s responsive care and consistent support could strengthen adolescents’ mental health and reduce their susceptibility to negative emotions [15]. Abundant studies have supported this contention by showing that authoritative parenting is negatively associated with adolescent mental health problems [6,11]. For example, in a study of 500 Indonesian adolescents, Abubakar et al. found that both maternal and paternal authoritativeness were positively related to adolescent psychological wellbeing [38].

A growing number of longitudinal studies have shown that adolescent mental health problems positively predict delinquent behavior [39,40]. The general strain theory (GST) contends that negative emotions are a leading cause of delinquency [41,42]. According to the GST, negative emotions foster irritability, impatience, and explosiveness, which increase the probability of delinquency [43]. Delinquent involvement, in turn, places adolescents at higher risk for victimization [15].

Prior research has found that adolescents with internalizing problems are more likely to be victimized because they tend to manifest emotional symptoms, such as anger and aggression, that might irritate potential perpetrators [44]. Empirical studies have indicated that adolescents who are unable to regulate their emotions tend to provoke confrontation and conflicts with peers, either intentionally or unintentionally, which may render them susceptible to victimization [45]. In accordance with these studies, Baldry and Farrington found that adolescents suffering from mental health problems were more likely to solve problems in an emotion-oriented way, such as violence and aggression, which left them open to victimization [46].

Based on prior research, we expect that mental health problems act as a critical mediator linking authoritative parenting, juvenile delinquency, and victimization. We therefore hypothesize the following:

**Hypothesis** **5** **(H5):** *Authoritative parenting decreases adolescents’ mental health problems, which in turn reduces the likelihood of juvenile delinquency.*

**Hypothesis** **6** **(H6):** *Authoritative parenting decreases adolescents’ mental health problems, leading to a lower likelihood of crime victimization.*

### 1.4. The Overlap between Delinquency and Crime Victimization

Victimization and delinquency rarely take place independently [47]. Abundant research has shown that offenders and victims are often the same group of people. The offender–victim overlap can be explained by the routine activity theory [48] and lifestyle exposure theory [49], both of which attribute the overlap to the shared routine activities and lifestyles of offenders and victims. Adolescents’ delinquent lifestyles increase their contact with potential offenders who may pose a threat to their safety, thus elevating their likelihood of victimization. Previous longitudinal studies have confirmed the comorbidity of delinquency and victimization by showing that ones’ own delinquent behavior is a strong predictor of their subsequent crime victimization [10,47]. In a comprehensive review of 37 studies examining the association between delinquency and crime victimization, Jennings, Piquero, and Reingle also found that those committing delinquent behavior were more likely to be victimized [50].

On the basis of studies reviewed in this section, we propose the following hypothesis:

**Hypothesis** **7** **(H7):** *Juvenile delinquency increases the likelihood of subsequent crime victimization.*

## 2. The Current Study

Victimological studies have predominantly focused on victim’s lifestyles and exposure to criminogenic opportunities as predictors of crime victimization. Relatively less is known about the role of family processes, such as authoritative parenting, in shaping adolescent vulnerability to crime victimization. Furthermore, previous studies on the offender–victim nexus have rarely related it to parenting factors. Considering the importance of authoritative parenting in facilitating child development and socialization, it is worthwhile to explore how authoritative parenting influences adolescents’ likelihood of crime victimization and the mechanisms through which such influence takes place. In addition, previous studies of parenting styles, juvenile delinquency, and victimization have mostly adopted cross-sectional designs, making it difficult to draw any causal inference about the relationships among key elements in the processes. 

**Figure 1:**
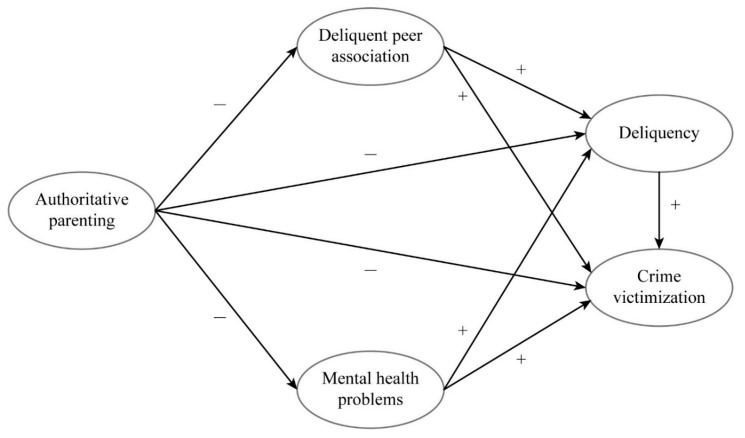
Theoretical Framework.

The current study aims to address the gaps in existing literature by constructing and testing a theoretical model that links authoritative parenting to juvenile delinquency and crime victimization, and by identifying the mechanisms that enable the processes. Additionally, the present study takes a step further to examine the connection between delinquency and crime victimization. To the best of our knowledge, this is the first empirical study that employs longitudinal data to examine how authoritative parenting directly and indirectly influences crime victimization. The theoretical model of this study is illustrated in Figure 1. 

## 3. Materials and Methods

### 3.1. Data

The current study used data collected from a two-wave longitudinal research project on family processes and delinquency. This study underwent a human subject review and was approved by the Research Ethics Committee of University of Macau on 19 December 2014 (Project identification code is MYRG2014–00120-FSS). The first wave of data was collected in one of the largest metropolitan areas in China in 2015 and the second wave of the survey took place one year after the baseline survey. The research site had been a major city in China before the country opened up its economy to the world in the late 1970s, but it has developed into a highly populated and diverse regional urban center in recent years with mixed urban and suburban districts. It is now home to 30 million people, including millions of migrant workers and ethnic minorities. 

To ensure the representativeness of the sample, we randomly selected eligible participants for the study using a three-stage stratified probability proportionate to size sampling procedure. In the first stage, we randomly selected 3 districts to study, including 2 urban districts, and 1 suburban district. In the second stage, 1 suburban middle school, 1 urban middle school, 1 suburban high school, and 1 urban high school were randomly sampled within each district, which yielded a total of 12 schools. In the third stage, in each sampled school, we proportionately selected a random number of classes in the seventh, eighth, tenth, and eleventh grades. Considering that ninth and twelfth graders (the final years of middle and high school) would graduate before the start of the second wave of the survey, we did not include them in the baseline survey. 

We contacted the sampled schools to seek their support and cooperation for the study. If the sampled school or class refused to participate in this survey, we randomly selected a replacement school or class until the sample size was reached. Once we obtained the cooperation of a school, we visited the school to introduce our study and sample the students. We provided the schools with the written informed consent forms for both the students and their parents. The forms contained information about the background and objectives of the study, the survey procedures, and a summary of the questions about which the students will be asked. In addition, the consent forms clearly state that the participation in this study is entirely voluntary, and the privacy and confidentiality of the respondents will be strictly protected. We also asked the students to provide contact information if they agreed to be followed up in the second wave. Only students who agreed to participate in both waves of the study and whose parents signed a consent form were included in the current study, which yielded 1300 eligible participants. A paper-and-pencil survey was administered to the sampled students. In the following year (2016), we conducted the second wave of survey in the same schools with the same class of students. The response rates for the Wave 1 and Wave 2 surveys were 97.20% and 96.73%, respectively. Additionally, 234 participants who had missing values on study variables, including the non-respondents, were excluded in the analyses, resulting in a final sample of 1066. 

### 3.2. Measurement

The key variables in this study, including authoritative parenting, mental health problems, delinquent peer association, delinquency, and crime victimization were measured using standard instruments with demonstrated validity and reliability. Crime victimization was measured by data collected in Wave 2 (W2), while all other variables were measured using data collected in Wave 1 (W1). We also included a wide range of control variables in the analyses, including age, gender, parents’ educational level, and family monthly income. 

*Authoritative parenting*. Prior research has identified three dimensions of authoritative parenting, including acceptance-involvement, behavioral supervision, and psychological autonomy [15,51,52]. In the current study, a total of 21 items corresponding to the three dimensions based on the National Longitudinal Survey of Youth 1997 [53] formed a scale measuring authoritative parenting. Acceptance-involvement was measured by 9 items asking the adolescents how often their parents behave in a caring, loving, and involved manner (e.g., “When I feel sad, I can get comfort from him/her.”; Cronbach α = 0.93). Behavioral supervision was measured by 10 items asking the adolescents how often their parents set rules and exercise monitoring on them (e.g., “He/she sets the limits on how late you stay out at night.”; Cronbach α = 0.89). Psychological autonomy was measured by 3 items asking the adolescents how often their parents adopt democratic discipline in childrearing and encourage them to express individuality (e.g., your father/mother encourage you to be involved in family decisions; Cronbach α = 0.81). Factor loading of the 21 items measuring the three dimensions on the construct of authoritative parenting all exceeded 0.5. The respondent was asked to rate his or her father/father figure and mother/mother figure separately on a five-point scale ranging from 1 (never) to 5 (always). A Father/mother figure is defined as an older man/woman who the student treats like a father/mother, especially by asking for his/her advice, help, or support. Ratings of both a father/father figure and mother/mother figure were combined. The mean scores of the items of each dimension were used as observed indicators of the latent variable of authoritative parenting in SEM analysis.

*Mental health problems*. Mental health problems were measured by Middle-School-Students Mental Health Inventory (MMHI) developed by Wang and his colleagues [54]. The reliability and validity of MMHI has been well established in various empirical studies among Chinese adolescents [54]. MMHI was composed of a total 10 subscales, each of which had 6 items. In the present study, we only selected five subscales to measure 5 psychological disorder symptoms including depression (Cronbach α = 0.82), anxiety (Cronbach α = 0.88), hostility (Cronbach α = 0.84), paranoid ideation (Cronbach α = 0.82), and interpersonal strain (Cronbach α = 0.76). MMHI asked how often (1 = never to 5 = always) the adolescents have the stated psychological disorder symptoms. We calculated the mean scores of each of the 5 subscales and used them as observed indicators of the latent variable of mental health problems in SEM analysis.

*Delinquent peer association*. Delinquent association was measured by a 5-item scale developed by Stouthamer-Loeber et al. [55]. The 5-item scale asked the respondents how many of their friends had been involved in 5 types of delinquent behaviors, including fighting, stealing, vandalism, threatening others, and joining a gang. Each item was rated on a five-point scale ranging from 1 (none) to 5 (all), with higher scores indicating more delinquent association. The scores of the 5 items were used as observed indicators of the latent variable of delinquent association in SEM analysis. Cronbach α value of the 5 items was 0.77.

*Delinquency*. Delinquency was measured by 18 dichotomized items adopted from the National Youth Survey [56]. The 18-item scale asked the respondents whether they had committed any of the delinquent acts within a year, including consuming alcohol, smoking, using drugs, selling drugs, fighting with others, threatening someone with weapons, hurting someone with weapons, running away from home, stealing something worth less than 500 RMB [USD70], snatching property from others, committing vandalism, bringing a knife to school, beating or threatening to beat someone, seriously injuring someone, stealing something worth more than 500 RMB [USD70], robbery, joining gang. The response categories for each item were ‘1’ for ‘Yes’ and ‘0’ for ‘No’. Summation of the 18 items formed the measurement of delinquent behavior. A higher score indicated more delinquent involvement. The range of delinquency was from 0 to 18.

*Crime victimization*. Crime victimization was measured by 6 dichotomized items asking respondents whether they had been exposed to 6 types of crime victimization within a year, including being stolen, being robbed, being threatened with weapons, being injured with weapons, being beaten (slapped, choked, kicked), and being severely injured. The response categories for each item were ‘1’ for ‘Yes’ and ‘0’ for ‘No’. Summation of the 6 items formed the measurement of crime victimization. A higher score indicated more exposure to crime victimization. The range of crime victimization was from 0 to 6.

*Control variables.* Control variables involved in the theoretical model included age, gender, parent’s educational level, and family monthly income. Age was an interval variable measured by year. Gender was a dichotomous variable, with ‘0’ for ‘male’ and ‘1’ for ‘female’. Parent’s education level was measured by asking the respondents to rate paternal education level and maternal education level separately on a 4-point scale ranging from 1 (primary school or less) to 4 (undergraduate education and above). The categorization of education level was based on national classifications, which were primary school, secondary school (middle and high school), junior college, undergraduate education, and above. Family monthly income was reported by the respondents on 4 categories ranging from 1 (less than RMB 1000 [USD140]) to 4 (more than RMB 9000 [USD1260]). As there was no standard classification of income categories on the national level, the categorization of income was based on the low-income cut-off of the city where we conducted the study, which was around RMB 1000 [USD140] in 2015.

### 3.3. Analytical Approach

A descriptive analysis was firstly applied to provide an overview of the sample. Pairwise Pearson correlation was then conducted to assess the bivariate correlations of the studied variables. Lastly, structural equation modelling (SEM) analysis was performed to test the research hypotheses. Direct, indirect, and total effects of authoritative parenting, mental health problems, and delinquent peer association on both delinquency and victimization were also calculated to identify the mechanisms through which authoritative parenting is related to delinquency and victimization. Stata 15.1 was used to estimate the SEM model. Chi square, root mean square error of approximation (RMSEA) comparative fit index (CFI), and the Tucker–Lewis index (TLI) were used as the good of fit indexes to assess the performance of SEM. A *p* value less than 0.05 was taken as significant in the current study. Case-wise deletion was applied to handling the item missingness and non-response. Clustered robust standard estimator was used to estimate the standard error of the SEM model so as to adjust the clustering nature of the sample. 

## 4. Results

Table 1 provides the descriptive statistics of exploratory, outcome, and control variables. As shown in the table, 51% of the respondents were female, and their average age was approximately 14 years old. Nearly three quarters of the respondents’ family income were between 1000 and 5000 RMB (74.49%) and 18.20% of respondents’ family income were between RMB 5001 and 9000. Only 2.25% of the respondents’ family income were below RMB 1000 and 5.07% of their family income were larger than 9000. Respondents’ parents received an average slightly less than high school education. In addition, the respondents reported receiving moderate levels of parental acceptance, autonomy, and supervision, indicating their parents overall adopted modest levels of authoritative parenting. As for the mental health problems, the respondents scored above 2 in all but one category. According to Wang et al., an average score higher than 2 in any category indicated at least a mild level of mental illness in the category [54]. The results suggested that the adolescents in the sample might have experienced some levels of depression, anxiety, paranoid ideation, and interpersonal strain. As a whole, the respondents reported relatively low levels of delinquent peer association and delinquency. Further, the respondents experienced less than one type of victimization. The average rate of victimization was 32.46%. 

Table 2 lists the pairwise Pearson correlations between three dimensions of authoritative parenting, mental health problems, delinquent peer association, delinquent behavior, and victimization. As shown in Table 2, acceptance, autonomy, and supervision were all significantly and negatively associated with delinquency and victimization. In addition, these dimensions of authoritative parenting were negatively correlated with delinquent peer association, while the latter was found to be positively associated with both delinquency and victimization. Therefore, delinquent peer association could be a mediator between the authoritative parenting and the delinquency/victimization nexus. Acceptance, autonomy, and supervision were also all negatively associated with mental health problems and mental health problems were positively associated with both delinquency and victimization. Finally, delinquency and victimization were shown to be positively associated.

**Figure 2:**
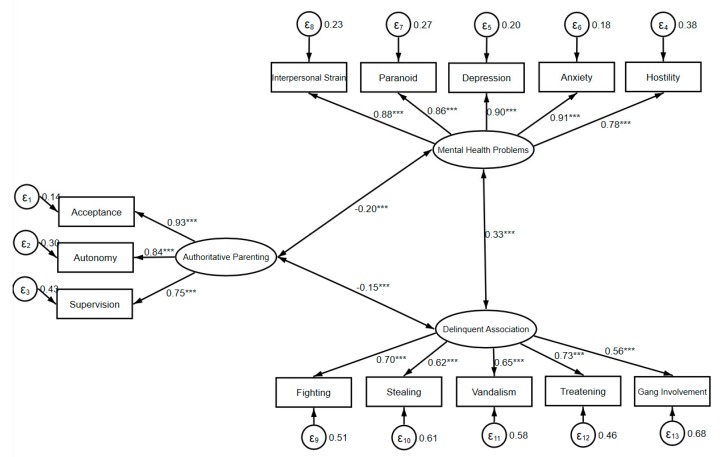
X^2^ = 310.888, d.f. = 62, root mean square error of approximation (RMSEA) = 0.062, comparative fit index (CFI) = 0.968, Tucker–Lewis Index (TLI) = 0.960. All of the coefficients are standardized.

A measurement model was firstly estimated to assess whether the three indicators of authoritative parenting, five indicators of mental health problems, and five indicators of delinquent peer association fit the data. As shown in Figure 2, the overall goodness of fit indexes of the measurement model were acceptable (X^2^ = 310.888, d.f. = 62, RMSEA = 0.062, CFI = 0.968, TLI = 0.960). The factor loadings of the indicators were large than 0.5. Authoritative parenting was also shown to be negatively and significantly associated with mental health problems (r = −0.20, *p* < 0.001) and delinquent association (r = −0.15, *p* < 0.001). Mental health problems were positively related to delinquent association (r = 0.33, *p* < 0.001).

**Figure 3:**
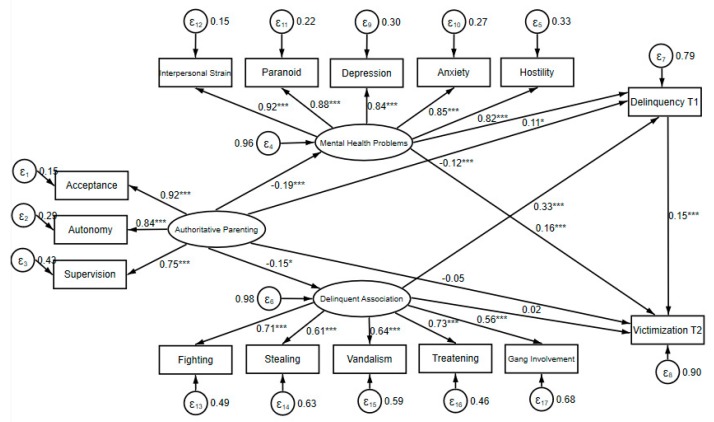
X^2^ = 482.540, d.f. = 143, root mean square error of approximation (RMSEA) = 0.047, comparative fit index (CFI) = 0.960, Tucker–Lewis Index (TLI) = 0.949. All of the coefficients are standardized. Control variables (not shown) include gender, age, family monthly income, paternal, and maternal education level.

Structure equation modeling analysis was applied to investigate the mediating effect of mental health problem and delinquent association on delinquency and victimization while controlling for gender, age, family income, and paternal and maternal education. As illustrated in Figure 3, the model overall goodness of fit was acceptable (X^2^ = 482.540, d.f. = 143, RMSEA = 0.047, CFI = 0.960, TLI = 0.949). The factor loadings of all the indicators indicated a good fit of the measurement part of SEM. For example, the factor loadings of all the three dimensions of authoritative parenting exceeded 0.75. Similar statistics of the five indicators of mental health problems were also all higher than 0.82. The factor loadings of the five indicators of delinquent peer association, although generally lower than those of authoritative parenting and mental health problem, were all larger than 0.55. 

Structural part of the SEM provided further insights about the mechanisms through which authoritative parenting was related to delinquency and victimization. Authoritative parenting was negatively related to delinquency. However, the direct effect of authoritative parenting on victimization was not significant, suggesting that the influence of authoritative parenting on victimization may be completely mediated by other variables. Authoritative parenting was significantly related to mental health problems and delinquency peer association. Mental health problems were positively related to delinquency and victimization. In a similar vein, delinquent peer association was also found to be significantly related to delinquency and victimization. Finally, delinquency was also found to be significantly related to victimization.

A further decomposition of direct, indirect, and total effect of authoritative parenting, mental health problems, and delinquent peer association on delinquency and victimization was given in Table 3. Thirty-seven percent of the association (−0.07/−0.19 = 0.37) between authoritative parenting and delinquency could be explained by the mediating effect of mental health problem and delinquent peer association. Although it did not have a significantly direct effect on victimization, authoritative parenting appeared to significantly reduce victimization through mitigating mental health problems, weakening association with delinquent peers, and decreasing delinquent involvement. Moreover, delinquency mediated the effects of mental health problems and delinquent peer association on crime victimization through two different pathways. Specifically, delinquency partially mediated the effect of mental health problems on victimization but fully mediated the effect of delinquent peer association on victimization. 

## 5. Discussion

The current study seeks to explore how authoritative parenting influences adolescent crime victimization. Although previous studies have recognized the important role of authoritative parenting in fostering child and adolescent development, few studies have examined the relationship between authoritative parenting and crime victimization. Moreover, we know little about the mechanisms through which authoritative parenting may potentially influence adolescents’ exposure to crime victimization. The objectives of the current study were to bridge these knowledge gaps. Through the analysis of the longitudinal data collected from a probability sample in China, the current study found an overall negative relationship between authoritative parenting and adolescent crime victimization. Further, the study showed that much of the relationship between authoritative parenting and crime victimization were indirect, mediated by adolescents’ delinquent peer association and mental health problems. Specifically, authoritative parenting weakened delinquent peer association and mitigated mental health problems, resulting in a lower likelihood of crime victimization. These findings were in line with prior research suggesting that authoritative parenting is conducive to the formation of conventional peer relationships [8]. Association with prosocial groups decreases adolescents’ exposure to high-risk situations, thereby reducing the likelihood of delinquency and victimization. In addition, prior research also indicated that authoritative parenting inhibits the development of mental health problems among adolescents, which in turn decreases their involvement in delinquent behavior and future crime victimization. 

Delinquency is another variable significantly mediating the relationship between authoritative parenting and crime victimization, especially with regard to the mechanisms involving delinquent peer association. Delinquency takes on the mediating role in three ways. First, it played a central role directly connecting authoritative parenting to crime victimization. Furthermore, it mediated the influences of the two other variables that link authoritative parenting to victimization, namely, delinquent peer association and mental health problems. While delinquency only partially mediated the pathway from authoritative parenting to crime victimization through mental health problems, it fully mediated the effect of delinquent peer association on victimization. Previous research has recognized the comorbidity of delinquency and victimization [10,29,47]. Our study takes a step further to demonstrate how delinquency operates in conjunction with other social and psychological mechanisms to shape the likelihood of crime victimization. 

Overall, our results were consistent with the growing body of studies showing that the parenting style that combines support and control is effective in reducing adolescent conduct problems [8,21,22,57]. Authoritative parenting is conducive to the establishment of a positive parent–child relationship, which facilitates children’s open communication with parents, thereby enhancing parents’ ability to identify potential risks that children may face and intervene when necessary [58]. A positive relationship between parents and children could inhibit delinquent behavior since children are more likely to conform to rules and follow suggestions when they are embedded in a warm family environment [16]. As a result, adolescents growing up in authoritative families are at lower risk for delinquency and crime victimization. 

The findings of the present study carry important implications for developing informed prevention and intervention strategies aimed at reducing juvenile delinquency and crime victimization. Research has indicated that 62% of adolescents who have encountered victimization shared the experience with their parents [59], suggesting that parents may have a great chance to recognize and intervene in adolescent victimization. In light of this, prevention and intervention programs should empower parents to adopt appropriate strategies, such as improving adolescents’ social networks, reducing their association with delinquent peers, strengthening their ability of emotional regulation, and preventing them from becoming involved in delinquent behavior, as means to protect adolescents from repeated victimization in the future. Considering authoritative parenting plays a critical role in inhibiting delinquency and crime victimization, parenting education programs should enhance parents’ awareness of the need for a proper balance between providing support and imposing control. Parents should learn to make effective efforts to monitor their children’s daily activities and peer association while providing responsive care and support that are critical to the development of psychological wellbeing and social skills. Such programs would also promote a positive parent–child relationship, which may further foster adolescents’ psychosocial development. Furthermore, our results show that adolescents who are involved in delinquency have a much higher likelihood of being victimized, suggesting that lifestyle factors strongly influence victimization. Based on this finding, prevention programs should help adolescents recognize and avoid risk factors related to delinquent lifestyles, thereby preventing them from future victimization.

Despite the contributions the current study made to the research on parenting and adolescent victimization, several major limitations are worth noting. First of all, the sample generated for this study was selected from secondary schools in one city in China. Students who dropped out of school and those absent from class on the dates of the survey were not included, which might constrain the generalizability of the findings of this study to the larger population of adolescents. It is also unclear whether the patterns observed in this study can be replicated and generalized to other cities in China. Second, due to lack of data, this study did not control for situational variables, such as neighborhood safety and school environment. As these situational factors may influence adolescents’ exposure to delinquency and victimization, the omission of these variables may undermine the accuracy of our findings. Third, most of the measures used in this study were based on the children’s perceptions using self-report data. Although children’s perceptions represent a reasonable way to measure parenting styles and their impact on children [60], they may be biased by children’s own interpretation of the behavior or event in question. Fourth, as this study focused specifically on authoritative parenting, it did not include measures of other parenting styles, such as indulgent parenting. The lack of attention to other parenting styles prevented us from drawing a robust conclusion about whether authoritative parenting is the most effective parenting style in inhibiting delinquency and victimization, especially in light of the emerging evidence showing that indulgent parenting is more likely to produce positive developmental outcomes than is authoritative parenting in some cultural settings [19,61]. To address these limitations, future research should use nationally representative data to validate the research findings. Furthermore, further studies should include the measures of other parenting styles as well as contextual variables such as school and community characteristics, which would gain a full understanding of parenting styles and the factors that influence juvenile delinquency and crime victimization. Additionally, future studies could consider incorporating reports of multi-informants such as parents, teachers, and peers, in order to capture the full range of experiences of parenting practices, juvenile delinquency, and victimization.

## 6. Conclusions

In conclusion, the present study demonstrated that authoritative parenting acted as a protective factor against juvenile delinquency and crime victimization. Additionally, delinquent peer association and mental health problems operated as critical mediating mechanisms linking authoritative parenting to juvenile delinquency and crime victimization. Specifically, adolescents exposed to higher levels of authoritative parenting were less likely to associate with delinquent peers and develop mental health problems, which in turn reduced their risk for delinquency and subsequent crime victimization. Finally, the results showed that juvenile delinquency played a key role in linking authoritative parenting to adolescent crime victimization. Authoritative parenting decreased the likelihood of delinquency, leading to a lessened possibility of future crime victimization. From a practical perspective, this study underscored the importance of developing family-based programs to target delinquent peer association, mental health problems, and juvenile delinquency in the prevention of adolescent crime victimization.

## Figures and Tables

**Table 1:** Descriptive analysis.

Variables	N	Mean/%	S.D.	Min	Max
*Demographic characteristics*					
Female	1066	0.51	0.50	0	1
Age (years)	1066	13.80	1.48	10	16
Family monthly income (RMB)					
Less than RMB 1000	24	2.25%			
RMB 1000–5000	794	74.49%			
RMB 5001–9000	194	18.20%			
More than RMB 9000	54	5.07%			
Paternal education level	1066	1.93	0.57	1	4
Maternal education level	1066	1.87	0.57	1	4
*Authoritative parenting*					
Acceptance	1066	3.31	0.78	1	5
Autonomy	1066	3.20	0.93	1	5
Supervision	1066	3.26	0.78	1	5
*Mental health problems*					
Depression	1066	2.08	0.80	1	5
Anxiety	1066	2.23	0.90	1	5
Hostility	1066	1.91	0.80	1	5
Paranoid ideation	1066	2.07	0.74	1	5
Interpersonal strain	1066	2.11	0.75	1	5
*Delinquent association, delinquency and victimization*					
Delinquent Peer Association	1066	1.24	0.44	1	5
Delinquency W1	1066	0.81	1.23	0	8
Victimization W2	1066	0.55	0.96	0	5

**Table 2:** Correlation coefficients matrix.

Variables	(1)	(2)	(3)	(4)	(5)	(6)	(7)	(8)	(9)	(10)	(11)
1. Acceptance	1										
2. Autonomy	0.78	1									
3. Supervision	0.70	0.63	1								
4. Depression	−0.20	−0.17	−0.17	1							
5. Anxiety	−0.15	−0.11	−0.11	0.85	1						
6. Hostility	−0.15	−0.13	−0.12	0.68	0.69	1					
7. Paranoid ideation	−0.15	−0.14	−0.14	0.73	0.76	0.74	1				
8. Interpersonal strain	−0.15	−0.14	−0.14	0.78	0.79	0.69	0.78	1			
9. Delinquent association	−0.13	−0.08	−0.12	0.25	0.23	0.30	0.28	0.26	1		
10. Delinquency W1	−0.15	−0.12	−0.18	0.23	0.19	0.28	0.18	0.21	0.35	1	
11. Victimization W2	−0.11	−0.13	−0.10	0.21	0.17	0.20	0.20	0.19	0.15	0.23	1

Note: All the correlation coefficients reach *p* < 0.001 level.

**Table 3:** Direct, indirect, and total effects of authoritative parenting, mental health problems, and delinquent peer association.

Variables	Authoritative Parenting	Mental Health Problems	Delinquent Association	Delinquency W1
Delinquency W1				
Direct	−0.12 ***	0.11 *	0.33 ***	
Indirect	−0.07 **	--	--	
Total	−0.19 ***	0.11 *	0.33 ***	
Victimization W2				
Direct	−0.05	0.16 ***	0.02	0.15 ***
Indirect	−0.06 ***	0.02 *	0.05 **	--
Total	−0.12 ***	0.18 ***	0.07 *	0.15 ***

Note: All the coefficients are standardized. *** *p* < 0.001, ** *p* < 0.01, * *p* < 0.05.
